# Supplementary material for: Local frustration determines loop opening during the catalytic cycle of an oxidoreductase
Source: eLife. 2020 Jun 22;9:e54661. doi: 10.7554/eLife.54661 (PMC7347389; doi:10.7554/eLife.54661)
Supplement: Figure 2—figure supplement 1—source data 1. [file elife-54661-fig2-figsupp1-data1.docx]

**Figure 2 – figure supplement 1 - source data 1. Quality factors (Q) and alignment tensor parameters (D_a_, R, θ, φ, ψ) obtained from fits of experimental RDCs to X-ray structures and**

**from analysis of the MD trajectories.**

| **nDsbD_ox_ using the 1L6P X-ray structure** | | | | | | | |
| --- | --- | --- | --- | --- | --- | --- | --- |
| **Region for which RDCs were fitted** | **Number of residues fitted** | **Q** | **D_a_** | **R** | **θ** | **φ** | **ψ** |
| 8-122 | 87 | 0.34 | -15.7 | 0.56 | 83.6 | 86.5 | 70.2 |
| 12-122 | 83 | 0.26 | -16.4 | 0.61 | 83.1 | 85.1 | 71.0 |
| 12-122  w/o 106/108^*^ | 81 | 0.24 | -16.8 | 0.61 | 83.7 | 84.8 | 71.7 |
| core | 46 | 0.21 | -17.7 | 0.61 | 84.7 | 83.2 | 72.9 |
| active site | 14 | 0.15 | -15.7 | 0.56 | 80.6 | 88.6 | 67.2 |
| **1 μs MD simulation of nDsbD_ox_** | | | | | | | |
| 8-122 | 87 | 0.26 |  |  |  |  |  |
| **nDsbD_red_ using the 3PFU X-ray structure** | | | | | | | |
| 8-125 | 90 | 0.38 | -15.2 | 0.56 | 85.2 | 88.8 | 105.3 |
| 12-122 | 87 | 0.29 | -16.5 | 0.57 | 85.5 | 91.1 | 105.5 |
| 12-122  w/o 106/108^*^ | 85 | 0.19 | -17.5 | 0.57 | 85.2 | 92.3 | 106.3 |
| core | 46 | 0.17 | -17.5 | 0.61 | 85.0 | 91.7 | 108.6 |
| active site | 16 | 0.16 | -18.6 | 0.52 | 88.4 | 93.9 | 106.2 |
| **1 μs MD simulation of nDsbD_red_** | | | | | | | |
| 8-125 | 90 | 0.23 |  |  |  |  |  |

^*^106/108 were excluded due to crystal contacts in the X-ray structures which may distort the conformation leading to poor fits; core means α1, β-sandwich and tight turn(s); active site means α2, β-strands but not loop (69-71).
