# Supplementary material for: Local frustration determines loop opening during the catalytic cycle of an oxidoreductase
Source: eLife. 2020 Jun 22;9:e54661. doi: 10.7554/eLife.54661 (PMC7347389; doi:10.7554/eLife.54661)
Supplement: Figure 2—figure supplement 2—source data 1. [file elife-54661-fig2-figsupp2-data1.docx]

**Figure 2 – figure supplement 2 - source data 1. Model-free analysis of ^15^N relaxation data for the cap loop and the flanking β-strands.**

|  | **nDsbD_ox_** |  |  | **nDsbD_red_** |  |  |
| --- | --- | --- | --- | --- | --- | --- |
|  | **{^1^H}-^15^N NOE** | **S^2^** | **τ_e_ (ps)** | **{^1^H}-^15^N NOE** | **S^2^** | **τ_e_ (ps)** |
| V64 | 0.67 | 0.87 | 83 +/- 62 | 0.77 | 0.87 |  |
| W65 | 0.70 | 0.86 | 54 +/- 20 | 0.76 | 0.87 |  |
| H66 |  |  |  | 0.83 | 1.0 |  |
| E67 |  |  |  |  |  |  |
| D68 | 0.59 | 0.86 | 108 +/- 85 | 0.77 | 0.85 |  |
| E69 | 0.50 | 0.89 | 334 +/- 121 | 0.70 | 0.87 | 63 +/- 18 |
| F70 | 0.61 | 0.91 | 246 +/- 136 | 0.77 | 0.93 |  |
| Y71 | 0.63 | 0.88 | 99 +/- 61 | 0.75 | 0.93 |  |
| G72 | 0.69 | 0.83 | 47 +/- 14 | 0.74 | 0.85 |  |
| K73 |  |  |  | 0.72 |  |  |
| S74 | 0.76 | 0.93 |  | 0.80 | 0.94 |  |
| E75 | 0.72 | 0.84 |  | 0.72 | 0.87 |  |
| I76 | 0.84 | 0.81 |  |  |  |  |
| Y77 | 0.78 | 0.82 |  | 0.87 | 0.86 |  |
