# Supplementary material for: Local frustration determines loop opening during the catalytic cycle of an oxidoreductase
Source: eLife. 2020 Jun 22;9:e54661. doi: 10.7554/eLife.54661 (PMC7347389; doi:10.7554/eLife.54661)
Supplement: Figure 6—figure supplement 1—source data 1. [file elife-54661-fig6-figsupp1-data1.docx]

**Figure 6 – figure supplement 1 - source data 1. Parameters from a multi-temperature analysis of the relaxation dispersion data.** Data were acquired at five temperatures (10, 15, 20, 25, 35 °C) at 500 MHz and at two temperatures (15 and 25 °C) at 750 MHz. The data were analyzed globally (Figure 6 – figure supplement 1). Peaks for residues 63, 64, 65, 70, 71, 73 and 74 were sufficiently resolved for peak intensity data to be obtained in all experiments. At the extreme temperatures of 10 ^o^C and 35 ^o^C peak intensities could not be determined with confidence for residues 66, 68, 72 and 75 because of peak overlap or exchange broadening. Similarly, peak intensities for residue 69, at 10 ^o^C and 15 ^o^C could not be determined. In total 73 CPMG relaxation dispersion curves were entered into the global analysis. A minimum of two temperatures and two magnetic fields were available for each residue allowing robust parameters to be obtained. The data were analyzed as described previously (Baldwin, Hilton, et al. 2011) using CATIA (Vallurupalli, Hansen, and Kay 2008). In CATIA, magnetization is simulated to match the experiment as closely as possible. To this end, the peak positions are entered into the program as well as the carrier frequency, allowing off resonance effects of the 180^o^ pulses to be accounted for. The difference between in-phase and anti-phase relaxation rates of coherences formed during the experiment was assumed to be 0.2 s^-1^ (Vallurupalli, Hansen, and Kay 2008). The populations of each state and rates of interconversion were assumed to follow Arrhenius behavior and chemical shifts were assumed to have a linear temperature dependence.

**A. Enthalpy and entropy parameters obtained from global fitting***

| ΔH_GE_ | -45 ± 6 kJ mol^-1^ |
| --- | --- |
| ΔS_GE_ | -180 ± 22 J mol^-1^ K^-1^ |
| ΔH^‡^_G<E_ | 38 ± 7 kJ mol^-1^ |
| ΔS^‡^_G<E_ | 74 ± 25 J mol^-1^ K^-1^ |
| ΔH^‡^_G>E_ | -6.80 ± 0.14 kJ mol^-1^ |
| ΔS^‡^_G>E_ | -105 ± 43 J mol^-1^ K^-1^ |

*The thermodynamic/activation parameters are defined as ΔX_GE_=X_E_-X_G_ (X=H, S) with E and G the excited and ground states, respectively. ΔX^‡^_G>E_ and ΔX^‡^_G<E_ refer to the forward and reverse reaction activation parameters, respectively. Errors in all parameters were obtained from a bootstrapping analysis where individual residues were sampled with replacement in all of the 7 combinations of temperature and magnetic field, prior to fitting. The resulting parameters were grouped into a histogram whose mean and standard deviation reflect the expectation value and uncertainty in each measurement (Press et al. 1988). Owing to large extrapolations in temperature inherent in the method, the uncertainty in the activation entropies (ΔS^‡^) was relatively high.

**B. Thermodynamic and kinetic parameters at each temperature from global fits.****

| Temperature / ^o^C | 10 | 15 | 20 | 25 | 35 |
| --- | --- | --- | --- | --- | --- |
| ΔG (kJ mol^-1^) | 6.1 | 7.0 | 7.9 | 8.8 | 10.6 |
| ΔG^‡^_G>E_ (kJ mol^-1^) | 23.0 | 23.6 | 24.1 | 24.6 | 25.7 |
| ΔG^‡^_G<E_ (kJ mol^-1^) | 17.0 | 16.6 | 16.2 | 15.9 | 15.1 |
| K_eq_ | 0.076 | 0.054 | 0.040 | 0.029 | 0.016 |
| *k_GE_* (s^-1^) | 47.9 | 46.3 | 44.9 | 43.6 | 41.2 |
| *k_EG_* (s^-1^) | 629.7 | 847.7 | 1129.9 | 1492.1 | 2534.6 |
| *k_ex_* (s^-1^) | 677.6 | 894.0 | 1174.8 | 1535.7 | 2575.8 |
| P_E_ (%) | 7.06 | 5.18 | 3.82 | 2.84 | 1.60 |

**To illustrate the effect of the various entropy and enthalpy contributions to the reaction, the following are the expectation values of all thermodynamic and kinetic parameters at the temperatures studied. ΔG^‡^_G>E_ and ΔG^‡^_G<E_ refer to the forward and reverse reaction activation free energies, respectively. Owing to the negative enthalpy of formation, the forward rate constant, *k_GE_* decreases with temperature. P_E_ is the population of the excited state.

**C. Kinetic parameters from single temperature fits.*****

| Temperature / ^o^C | 15 | 25 |
| --- | --- | --- |
| *k_GE_* (s^-1^) | 37.7 ± 13.9 | 31.0 ± 2.1 |
| *k_EG_* (s^-1^) | 850.2 ± 123.5 | 1368.9 ± 210.5 |
| *k_ex_* (s^-1^) | 887.9 ± 135.6 | 1399.9 ± 205.4 |
| P_E_ (%) | 4.3 ± 1.1 | 2.2 ± 1.2 |

***The following parameters were determined from single temperature fits using data collected at two magnetic fields at 15 ^o^C or 25 ^o^C; these value are consistent with the results obtained from global analysis of the entire dataset.

**D. ^15^N |Δω| values at 25 ^o^C from CPMG relaxation dispersion data analysis.******

| **Residue** | **^15^N \|Δω\| (ppm) (25 °C)** |
| --- | --- |
| G63 | 0.67 ± 0.19 |
| V64 | 1.18 ±0.23 |
| W65 | 2.12 ± 0.43 |
| H66 | 0.72 ± 0.19 |
| D68 | 0.80 ± 0.18 |
| E69 | 4.13 ± 0.89 |
| F70 | 3.34 ± 0.85 |
| Y71 | 1.91 ± 0.39 |
| G72 | 1.05 ± 0.22 |
| K73 | 3.99 ± 0.99 |
| S74 | 1.96 ± 0.42 |
| E75 | 0.17 ± 0.02 |

****Errors in Δω were obtained from a bootstrapping analysis where individual residues were sampled with replacement in all of the 7 combinations of temperature and magnetic field, prior to fitting. The resulting parameters were grouped into a histogram whose mean and standard deviation reflect the expectation value and uncertainty in each measurement (Press et al. 1988). Only residues with a Δω value of greater than 1 ppm are included in Figure 7.

**References**

Baldwin AJ, Hilton GR, Lioe H, Bagneris C, Benesch JLP, Kay LE. 2011. Quaternary dynamics of αB-crystallin as a direct consequence of localised tertiary fluctuations in the C-terminus." *Journal of Molecular Biology* **413**:310-320. DOI: 10.1016/j.jmb.2011.07.017, PMID:21839749.

Press WH, Flannery BP, Teukolsky SA, Vetterling WT. 1988. *Numerical Recipes in C*. Cambridge, U.K. : Cambridge University Press.

Vallurupalli P, Hansen DF, Kay LE. 2008. Structures of invisible, excited protein states by relaxation dispersion NMR spectroscopy. *PNAS* **105**:11766-11771. DOI: 10.1073/pnas.0804221105, PMID: 18701719.
