# Supplementary material for: Local frustration determines loop opening during the catalytic cycle of an oxidoreductase
Source: eLife. 2020 Jun 22;9:e54661. doi: 10.7554/eLife.54661 (PMC7347389; doi:10.7554/eLife.54661)
Supplement: Figure 6—figure supplement 2—source data 1. [file elife-54661-fig6-figsupp2-data1.docx]

**Figure 6 – figure supplement 2- source data 1.** **Crystallographic data collection and refinement statistics for Δloop-nDsbD_ox_**

| **Unit Cell** | |
| --- | --- |
| Space Group | P2_1_ |
| Cell dimensions (Å) | *a* = 37.33, *b* = 81.34, *c* = 46.39, β=100.66^o^ |
| **Data Collection** | |
| Temperature (K) | 100 |
| Wavelength (Å) | 1.5418 |
| Resolution (Å) | 39.77 to 2.60 (2.74 to 2.60)* |
| <I/σ> | 5.4 (3.3)* |
| R_meas_ | 0.31 (0.38)* |
| Multiplicity | 3.1 (2.9)* |
| **Refinement** | |
| Refinement program | Phenix.refine |
| Number of unique reflections used | 6808 |
| Completeness (%) | 80.7 (79.0)* |
| R-factor (%) | 25.2 |
| R_free_ (%) | 29.2 |
| Average B (Å^2^) | 26.0 |
| r.m.s.d. bond lengths (Å) | 0.003 |
| r.m.s.d. bond angles (°) | 0.721 |
| **Ramachandran** | |
| Favoured | 211 (95.0%) |
| Allowed | 9 (4.1%) |
| Outliers | 2 (0.9%) |
| **Asymmetric unit contents** | |
| Polypeptide chains | 2 |
| No. of atoms: total/non-solvent | 1838/1779 (13 in alternative conformations) |

* values in parentheses are for corresponding highest resolution cell

R_sym_ = Σ |I_h_ – <I_h_>|/ Σ I_h_

R-factor = Σ (|F_obs_| - |F_calc_|)/ Σ |F_obs_|

r.m.s.d. = root mean square deviation
