# Supplementary material for: Local frustration determines loop opening during the catalytic cycle of an oxidoreductase
Source: eLife. 2020 Jun 22;9:e54661. doi: 10.7554/eLife.54661 (PMC7347389; doi:10.7554/eLife.54661)
Supplement: Figure 7—source data 1. [file elife-54661-fig7-data1.docx]

**Figure 7 – source data 1. Determination of the sign of the ^15^N chemical shift difference between the excited state and the ground state determined by comparison of HSQC and HMQC spectra collected at 750 MHz.***

|  | Δω_HSQC-HMQC_ (ppm) | Δω_HSQC-HMQC_ (Hz) | Sign of Δω_HSQC-HMQC_ |
| --- | --- | --- | --- |
| V64 | -0.00052 ± 0.00092 | -0.039 ± 0.070 | n.d. |
| W65 | +0.00901 ± 0.00237 | +0.684 ± 0.180 | positive |
| E69 | -0.03161 ± 0.00027 | -2.402 ± 0.020 | negative |
| F70 | +0.00494 ± 0.00110 | +0.375 ± 0.084 | positive |
| Y71 | -0.00384 ± 0.00055 | -0.292 ± 0.042 | negative |
| G72 | +0.00913 ± 0.00010 | +0.693 ± 0.007 | positive |
| K73 | +0.01983 ± 0.00067 | +1.507 ± 0.051 | positive |
| S74 | -0.00083 ± 0.00140 | -0.063 ± 0.106 | n.d. |

*Chemical shift differences were measured from three pairs of HSQC/HMQC spectra collected at 750 MHz using pulse sequences developed by Skrynnikov *et al*. (Skrynnikov, Dahlquist, and Kay 2002). For W65, E69, F70, G72 and K73, |Δω_HSQC-HMQC_| is found to be > 0.3 Hz, the cut-off value proposed by Skrynnikov *et al.* for confidently reconstructing the sign information. For Y71, |Δω_HSQC-HMQC_| is just below the cut-off value of 0.3 Hz; however, negative values of Δω_HSQC-HMQC_ are measured in all three repeats increasing the confidence in the sign information. By contrast, for V64 and S74 both positive and negative Δω_HSQC-HMQC_ values were measured and the sign cannot be determined with confidence. The signs determined refer to Δω = ω_excited state_ – ω_ground state_. In Figure 7, the sign of Δω has been reversed because the chemical shift differences plotted are between the ground state (nDsbD_ox_) and the excited state (Δω = ω_ground state_ – ω_excited state_).
