# Supplementary material for: Local frustration determines loop opening during the catalytic cycle of an oxidoreductase
Source: eLife. 2020 Jun 22;9:e54661. doi: 10.7554/eLife.54661 (PMC7347389; doi:10.7554/eLife.54661)
Supplement: Transparent reporting form [file elife-54661-transrepform.docx]

***eLife’s* transparent reporting form**

We encourage authors to provide detailed information *within their submission* to facilitate the interpretation and replication of experiments. Authors can upload supporting documentation to indicate the use of appropriate reporting guidelines for health-related research (see [EQUATOR Network](http://www.equator-network.org/%20)), life science research (see the [BioSharing Information Resource](https://biosharing.org/)), or the [ARRIVE guidelines](http://www.plosbiology.org/article/info:doi/10.1371/journal.pbio.1000412) for reporting work involving animal research. Where applicable, authors should refer to any relevant reporting standards documents in this form.

If you have any questions, please consult our Journal Policies and/or contact us: [editorial@elifesciences.org](mailto:editorial@elifesciences.org).

**Sample-size estimation**

- You should state whether an appropriate sample size was computed when the study was being designed
- You should state the statistical method of sample size computation and any required assumptions
- If no explicit power analysis was used, you should describe how you decided what sample (replicate) size (number) to use

Please outline where this information can be found within the submission (e.g., sections or figure legends), or explain why this information doesn’t apply to your submission:

Sample-size estimation is not relevant to the study we describe.

**Replicates**

- You should report how often each experiment was performed
- You should include a definition of biological versus technical replication
- The data obtained should be provided and sufficient information should be provided to indicate the number of independent biological and/or technical replicates
- If you encountered any outliers, you should describe how these were handled
- Criteria for exclusion/inclusion of data should be clearly stated
- High-throughput sequence data should be uploaded before submission, with a private link for reviewers provided (these are available from both GEO and ArrayExpress)

Please outline where this information can be found within the submission (e.g., sections or figure legends), or explain why this information doesn’t apply to your submission:

The error bars in the order parameters shown in Figure 2 (and Figure 2 – figure supplement 2 B/C and 3) were determined using procedures widely adopted in the NMR community. As indicated in the legend to Figure 2, Monte Carlo simulations were used for error estimation from the experimental data. The Monte Carlo method is described in the ‘NMR Spectroscopy’ section in Materials & methods. The same procedure was used for the errors for τ_e_ listed in Figure 2 – figure supplement 2 - source data 2. The analysis of errors for S^2^ derived from the MD simulations is described in the ‘Analysis of the MD simulations’ section in Materials & methods. This analysis used resampling of S^2^ from trajectory segments. This method was also used for the errors on the RDCs calculated from the MD simulations shown in Figure 2 – figure supplement 1 C/D and the errors on the order parameters shown in Figure 2 and Figure 2 – figure supplement 3.

The error bars for the heteronuclear NOEs shown in Figure 2 – figure supplement 2A were estimated from 500 Monte Carlo simulations using the baseline noise as a measure of the error in the peak heights, as described in the ‘NMR Spectroscopy’ section in Materials & methods.

The error bars for the relaxation dispersion data analysis shown in Figure 6D/E/F, Figure 7, Figure 6 – figure supplement 1A and Figure 6 – figure supplement 1 - source data 1 were determined using a bootstrap analysis and the program CATIA as described in the footnotes to Figure 6 – figure supplement 1 - source data 1.

The errors shown in Figure 7 – source data 1 for the chemical shift differences measured in HSQC versus HMQC spectra were obtained as the standard deviations from the mean of 3 independent measurements of chemical shift differences in the pairs of NMR spectra.

**Statistical reporting**

- Statistical analysis methods should be described and justified
- Raw data should be presented in figures whenever informative to do so (typically when N per group is less than 10)
- For each experiment, you should identify the statistical tests used, exact values of N, definitions of center, methods of multiple test correction, and dispersion and precision measures (e.g., mean, median, SD, SEM, confidence intervals; and, for the major substantive results, a measure of effect size (e.g., Pearson's r, Cohen's d)
- Report exact p-values wherever possible alongside the summary statistics and 95% confidence intervals. These should be reported for all key questions and not only when the p-value is less than 0.05.

Please outline where this information can be found within the submission (e.g., sections or figure legends), or explain why this information doesn’t apply to your submission:

Not applicable.

(For large datasets, or papers with a very large number of statistical tests, you may upload a single table file with tests, Ns, etc., with reference to sections in the manuscript.)

**Group allocation**

- Indicate how samples were allocated into experimental groups (in the case of clinical studies, please specify allocation to treatment method); if randomization was used, please also state if restricted randomization was applied
- Indicate if masking was used during group allocation, data collection and/or data analysis

Please outline where this information can be found within the submission (e.g., sections or figure legends), or explain why this information doesn’t apply to your submission:

Not applicable.

**Additional data files (“source data”)**

- We encourage you to upload relevant additional data files, such as numerical data that are represented as a graph in a figure, or as a summary table
- Where provided, these should be in the most useful format, and they can be uploaded as “Source data” files linked to a main figure or table
- Include model definition files including the full list of parameters used
- Include code used for data analysis (e.g., R, MatLab)
- Avoid stating that data files are “available upon request”

Please indicate the figures or tables for which source data files have been provided:

Not applicable.
